# Incremental Concentrations of Tacrolimus Eye Drops as a Strategy for the Management of Severe Vernal Keratoconjunctivitis

**DOI:** 10.3389/fphar.2022.798998

**Published:** 2022-03-25

**Authors:** Maan Abdullah Albarry, Mohit Parekh, Stefano Ferrari, Heba Mahmoud Eltahir, Ahmed M Shehata, Mohamed A Shaker, Hossein Mostafa Elbadawy

**Affiliations:** ^1^ Department of Ophthalmology, College of Medicine, Taibah University, Madinah, Saudi Arabia; ^2^ Institute of Ophthalmology, University College London, London, United Kingdom; ^3^ International Center for Ocular Physiopathology, Veneto Eye Bank Foundation, Venice, Italy; ^4^ Department of Pharmacology and Toxicology, College of Pharmacy, Taibah University, Madinah, Saudi Arabia; ^5^ Department of Pharmacology, Faculty of Pharmacy, Beni-Suef University, Beni-Suef, Egypt; ^6^ Pharmaceutics and Pharmaceutical Technology Department, College of Pharmacy, Taibah University, Madinah, Saudi Arabia; ^7^ Pharmaceutics Department, Faculty of Pharmacy, Helwan University, Cairo, Egypt

**Keywords:** Human cornea, keratoconjunctivitis sicca, tacrolimus, immunosuppressant, eye drops

## Abstract

**Purpose:** To assess the effect of different concentrations of tacrolimus eye suspension on the epithelium and stromal keratocytes of human corneas and investigate whether it can be safely used for severe cases of vernal keratoconjunctivitis (VKC).

**Methods:** Tacrolimus eye suspension was prepared in a range of concentrations of 0.005%, 0.01%, 0.05%, 0.1%, and 0.2%. Molecular analysis was performed *ex vivo* on human corneas (*n* = 18), obtained from the eye bank. Transparency and thickness of each cornea were measured while live/dead staining was performed using a triple labeling assay. An incremental concentration approach was then tested on three severe cases of VKC.

**Results:** All tested tacrolimus concentrations showed no significant changes in corneal thickness or transparency. In corneas treated with 0.1%, rare scattered dead cells were observed, while the folds of corneal surfaces were mostly viable, unlike concentrations higher than 0.1% and lower than 0.05%. Stromal cell densities were highest in the 0.1% tacrolimus treatment condition. Incremental concentrations of tacrolimus suspension were shown to significantly improve VKC cases, where the concentration used for each case depended on the severity of the case.

**Conclusions:** Topical administration of tacrolimus was not toxic to human corneal cells at all tested concentrations, and the 0.1% concentration has shown the best viability of the corneal tissue. Tacrolimus eye suspension was shown to be safe and effective for use in severe VKC and is proposed as a topical ocular immunosuppressant drug enabling clinicians to incrementally increase the drug concentration according to the clinical severity of the disease to achieve the optimal therapeutic response.

## Introduction

Tacrolimus is a macrolide lactone isolated almost 40 years ago from *Streptomyces tsukubaensis* (Kino et al., 1987). It was shown to have a potent immunosuppressive action. Thus, tacrolimus is currently used after organ transplantation to prevent transplant rejection. It functions by binding to a specific protein (FK506-binding protein) within the T-lymphocytes and inhibiting the activity of calcineurin, resulting in a cascade of inhibitory effects of interleukins, interferons, and tumor necrosis factor (TNF) required to initiate an immune response (Tamura et al., 1994). Its inhibitory effect on T-lymphocytes also prevents the release of inflammatory cytokines, which aid in taming the inflammatory responses (Zhai et al., 2011). Tacrolimus eye drops have been used after cornea transplantation procedures with positive clinical outcomes and evident improvement of visual functions (Yamazoe et al., 2014; Ghaffari et al., 2017). It is also used in ocular graft-versus-host disease (GVHD) with good efficiency and tolerability (Abud et al., 2016). For this purpose, tacrolimus is commonly prescribed along with other immunosuppressant drugs such as sirolimus (McAlister et al., 2000). In addition to its use for this purpose for years, tacrolimus is also used in ophthalmology to lessen the inflammation associated with dry eye syndrome (Moscovici et al., 2012; Moscovici et al., 2015). It was also used for uveitis (Kawashima et al., 1988), atopic blepharitis (Iwamoto et al., 1999), allergic conjunctivitis refractory to corticosteroids treatment (Zribi et al., 2009), Sjögren’s syndrome-associated dry eyes (Moscovici et al., 2015), and vernal and atopic keratoconjunctivitis (Vichyanond et al., 2004; Ohashi et al., 2010). Tremors and hypomagnesemia were the most prominent side effects of oral tacrolimus tested on a large population of kidney transplantation patients, whereas hypercholesterolemia and hypertension are minor side effects of the prolonged use of tacrolimus over a 6-month period (Margreiter 2002).

Vernal keratoconjunctivitis (VKC) is an allergic ocular disease that shows seasonal and patient-related variations in its severity and symptoms and appears in children, especially boys. The disease is commonly self-limiting in most cases and improves with age. However, persistent cases are potentially sight-threatening. In VKC cases, inflammatory cells are recruited, leading to the activation of CD4^+^ T-lymphocytes and increased levels of inflammatory cytokines (Metz et al., 1997; Leonardi 2002). Symptoms of this condition commonly include excessive eye secretions, photophobia, irritation or foreign body sensation, and a tendency to continuous eye rubbing. Upon clinical examination, giant cobblestone papillae are usually seen, accompanied by inflammatory discharges, shield ulcers, and corneal lesions due to continuous friction with the eyelid (Addis and Jeng 2018).

Immunosuppressive drugs are used to control long-term immune reactions in the eye, which may damage the cornea, necessitating the need for corneal transplantation. Tacrolimus was tested on the eye at a concentration of 0.1% to treat the ocular allergic condition (Fukushima et al., 2014), 0.03% for Sjögren syndrome (Moscovici et al., 2015), and 0.005% for vernal keratoconjunctivitis (VKC) among a range of allergic ocular disorders. Fortunately, minimal side effects, including mild irritation and stinging sensation, have been reported for the topical use of tacrolimus on the ocular surface thus far (Fukushima et al., 2014).

*Ex vivo and in silico* (*via* computer simulation programs) experimentation on human corneas represents a promising and relevant approach to assess the effect of drugs used topically on the eye (Platania et al., 2015). In the current work, a range of tacrolimus concentrations was tested on human corneas *ex vivo* before testing tacrolimus eye suspension on clinical cases on refractory VKC cases to assess its potential effect and optimal concentration for treating refractory VKC. This should provide a therapeutic solution for such cases with minimal adverse effects in contrast to orally administered tacrolimus.

## Materials and Methods

### Human Corneal Cultures

A total of 18 human corneas were used in this work. The whole corneal surface integrity was examined using a slit lamp microscope. Corneas were obtained from the Veneto Eye Bank Foundation, checked for epithelial integrity by staining with trypan blue (Sigma-Aldrich, Italy) and examining the corneas using an inverted light microscope. Trypan blue dye was added for 1 min, followed by three washes with phosphate-buffered saline (PBS) and counting the number of dead cells. Corneas with epithelial defaults showing more than 5% positive staining were excluded from this study. Corneas with histories of scars, stromal abnormalities, or major defects were also excluded. Corneas were randomly allocated to six groups, each of three corneas. Corneas were transferred from organ culture media into a deswelling medium (with dextran) in 12-well plates 1 day before starting the experiments to retain original thicknesses of corneas and were supplemented with enough medium to cover the whole surface of the cornea (submerged culture). This work was carried out in accordance with The Code of Ethics of the World Medical Association (Declaration of Helsinki) for experiments involving humans. Ethical approval and informed consent were obtained for experimentation with human subjects.

### Treatment of Human Corneas With Tacrolimus

To study the acute effect of tacrolimus treatment, tacrolimus capsules (Prograf^®^1 Mg; Astellas Pharma Inc., Tokyo, Japan) were used to prepare five different concentrations: 0.005%, 0.01%, 0.05%, 0.1%, and 0.2% in (PBS) and a frequent treatment schedule was adopted. On the day of the experiment, the culture medium level in each well of the 12-well plate was reduced to just cover up to the limbus of the cornea. The tacrolimus solution at the different prepared concentrations was applied to the corresponding corneas in the assigned groups using a dropper for 2 min, and the solution was maintained on the corneal surface for 10 min before washing three times with PBS. Corneas were incubated for 2 h, and treatment was repeated. Tacrolimus treatment was repeated five times at a 2 h interval. Control corneas were treated in the same manner with PBS only.

### Measuring Corneal Transparency and Thickness

By the end of the tacrolimus treatment, corneal transparency was measured by a specific corneal transparency device previously reported by our group (Parekh et al., 2014). The device functions by measuring light transmission through the cornea versus controls. Corneal transparency was measured three times for each cornea, and the average was scored using a system of scaling from 0 to 4, where 0 indicates no opacity, 1 slightly hazy, 2 moderately opaque, 3 severely opaque, and 4 for complete loss of transparency. Corneal thickness was measured using the optical coherence tomography (OCT) technique (Tomey, Japan) for each cornea before and after treatment to be used as an indicator of swelling/edema. After measuring the corneal thickness in three different central areas of the corneal button, swelling was scored on a scale of 0–2, where 0 indicates no swelling, 1 mild swelling, and 2 severe swelling (Yoeruek et al., 2008). Corneal swelling of 25% or more was considered pathological (Elbadawy et al., 2015). Percentage changes in the thickness and transparency of corneas were calculated from the original measurements taken before the treatment of corneas.

### Cell Viability Assay

Triple labeling was performed using Ethidium Homodimer (E, red) to label dead cells, Hoescht 33,342 (H, blue) to stain the nuclei, and calcein AM (C, green) to label the viable cells as previously described (Pipparelli et al., 2011). Corneas were washed in PBS, immersed in optimal cutting temperature compound (OCT) (Diapath, Italy), and then snap-frozen in the −80 C freezer for 30 min. Sections of 10 µm thickness were generated using conventional cryostat microtome (Leica, Milan, Italy). Menzel SuperFrost plus slides from Diapath (Italy) were used for collecting sections. 4 µL of Hoescht 33342 (H) (Thermo Fisher Scientific, Rochester, NY, United States), 4 µL of ethidium homodimer EthD-1 (E), and 2 µL of calcein AM (C) (LIVE/DEAD Viability/Cytotoxicity Kit, Thermo Fisher Scientific, Rochester, NY, United States) were mixed in 1 ml of PBS. 100 µL of the final solution was topically applied on the sections and incubated at room temperature in the dark for 30 min. Sections were incubated with PBS only for 30 min under the same conditions were used as a negative control. The sections were washed with PBS and mounted. Then they were examined in a blind fashion with a Nikon Eclipse Ti-E (Nikon, Burgerweeshuispad, Amsterdam) using NIS Elements software (Nikon) under ×100 magnification, where three random snapshots were taken for each section. Fluorescence intensity was quantified using ImageJ software (nih.gov/ij), where the integrated density representing the total count of pixels was determined for each image. The percentage was then calculated by dividing the average of the staining quantification values of each treatment condition over the staining quantification values of PBS control treatment (DP = Av(treatment quantification)/Av(control quantification).

### DAPI Nuclear Staining

Human corneas were rinsed twice in PBS and then incubated in freshly prepared 4% paraformaldehyde at 4 C˚ overnight. Samples were then washed in PBS, immersed in optimal cutting temperature compound (OCT) (Diapath, Italy), and then snap-frozen in a −80 C freezer for 30 min. Sections of 10 µm thickness were generated using conventional cryostat microtome (Leica, Milan, Italy). Menzel SuperFrost plus slides from Diapath (Italy) were used for collecting sections. A Vectashield^®^ mounting medium with DAPI was added, and slides were stored at −4 C. Laser confocal microscope (Carl Zeiss, Germany) was used to detect DAPI staining in a blind fashion. Quantification of fluorescence intensity was performed using ImageJ software (nih.gov/ij) as detailed previously.

### Statistical Analysis

Before the statistical analysis of *ex vivo* data, treatments and grouping of human corneas were unmasked. Data from three replicate measurements were used for independent *t*-tests. Statistical analysis was performed using GraphPad InStat software (GraphPad Software, Inc. United States). Tests were two-tailed, and the significance level was set at *p* < 0.05%.

## Results

### The Effect of Tacrolimus on Corneal Thickness

**FIGURE 1:**
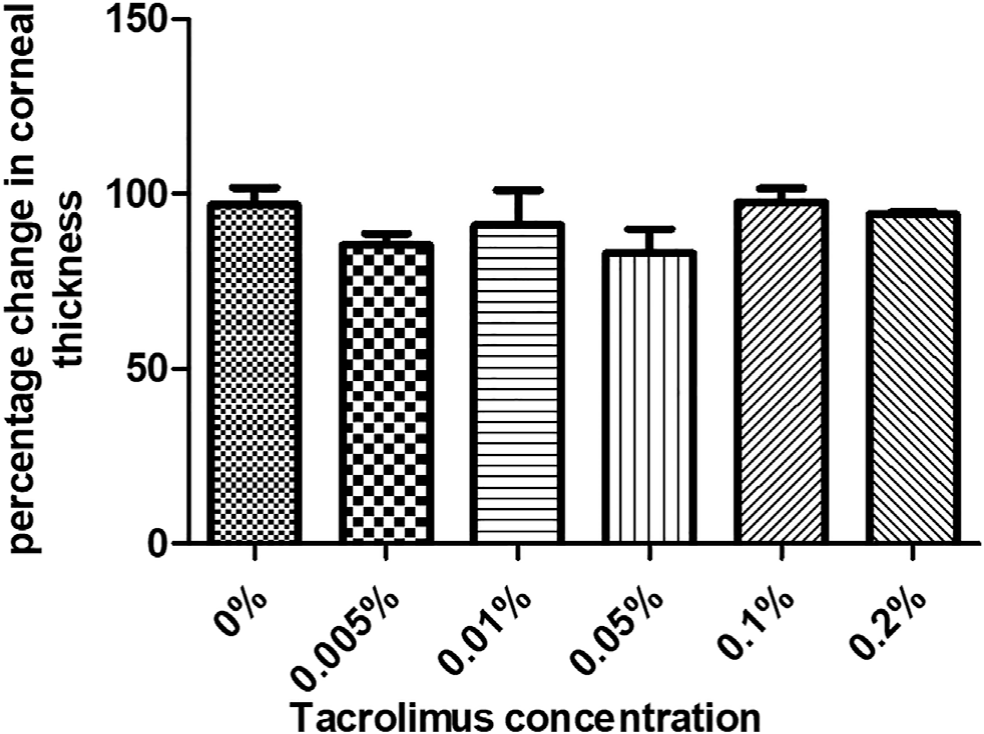
The effect of tacrolimus treatment on corneal thickness. Percentage change in corneal thickness from pretreatment measurements is represented by bars. Each bar shows the average from three corneas (*n* = 3). Error bars represent standard deviations.

The first parameter to evaluate the effect of tacrolimus concentrations on human corneas was to measure the thickness of the corneas treated with a range of concentrations. Corneal thickness was measured before and after treatment with the test agent. All treatment conditions showed a slight, statistically non-significant decrease in corneal thicknesses, indicating no pathological edema. Treatment with 0.005% tacrolimus eye drops resulted in a 14.6 ± 4% reduction in corneal thicknesses as compared to the pretreatment condition (*p* = 0.19). Doubling the concentration (0.01%) resulted in an average corneal deswelling of 8.89% ± 12.25 (*p* = 0.52) compared to the original thickness before treatment. The maximum corneal deswelling was 16.99% ± 8.52, noted in corneas treated with 0.05% tacrolimus solution. However, the change was insignificant (*p* = 0.24) and did not reach 25% (pathological swelling). Using highly concentrated solutions of 0.1% and 0.2%, tacrolimus showed the least thickness changes during the time course of the experiment, and the decreases compared to pretreatment thicknesses were 2.25 ± 4.74% and 5.82 ± 0.86%, respectively (*p* = 0.54 and *p* = 0.52, resp.). Tacrolimus did not cause edema or endothelial dysfunction (which causes fluid leakage into the cornea) at any of the tested concentrations, as indicated by the absence of corneal swelling. The corneal thickness score was 0 for all tested concentrations (Figure 1).

### The Effect of Tacrolimus on Corneal Transparency

The transparency of human corneas was measured before and after treatment with PBS or tacrolimus at different concentrations. Treatment with tacrolimus solution of 0.005% or 0.01% concentration resulted in a decrease in transparency by 2.94 ± 5.1% and 1.9 ± 3.29%, respectively. However, the change was significant only with 0.005% tacrolimus treatment (*p* = 0.45). Interestingly, 0.05% tacrolimus solution did not result in significant changes in corneal transparency (*p* = 0.21). At higher concentrations (0.1% and 0.2%), treatment with tacrolimus solution did not cause significant changes (*p* = 0.36 and *p* = 0.71, resp.).

**FIGURE 2:**
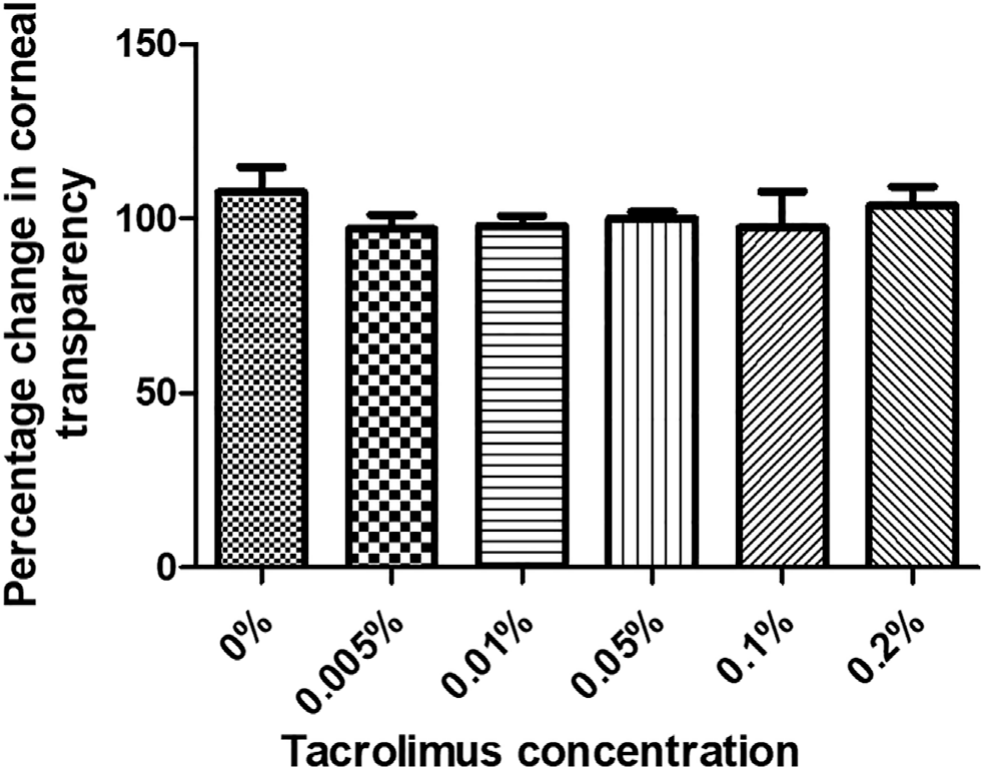
The effect of tacrolimus treatment on corneal transparency. Percentage change in corneal transparency from pretreatment measurements is represented by bars. Each bar shows the average from three corneas (*n* = 3). Error bars represent standard deviations.

No significant differences were noted when comparing corneas treated with tacrolimus concentrations higher than 0.005% with PBS or with other treatment concentrations. Tacrolimus eye drops did not affect corneal transparency at any of the tested concentrations as the corneal opacity score was 0 for all tested concentrations (Figure 2).

### Corneal Viability After Treatment With Tacrolimus

**FIGURE 3:**
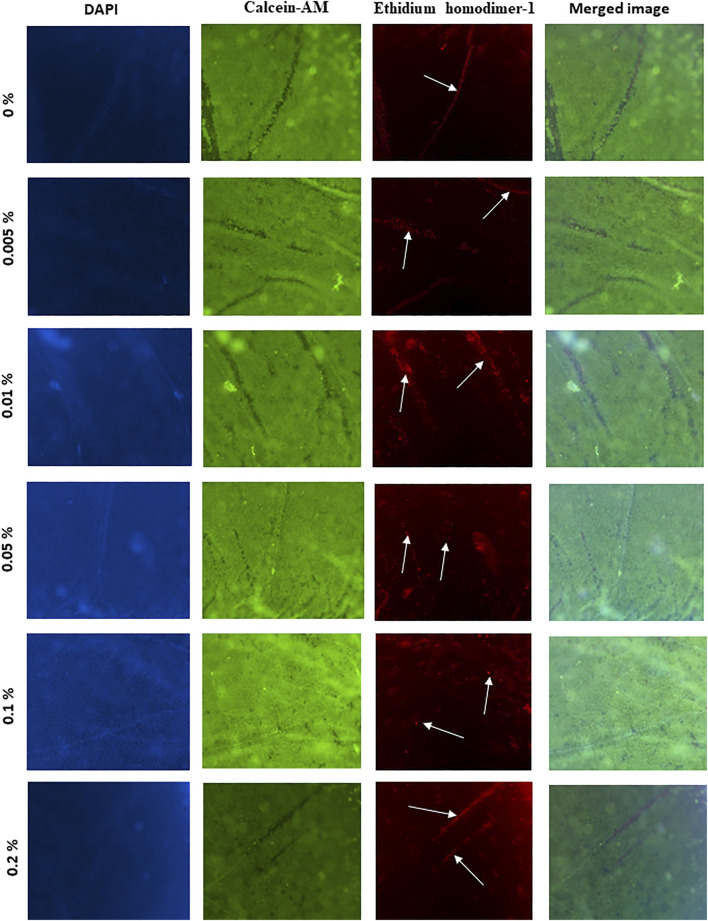
The effect of tacrolimus on epithelial cell viability. Concentrations of tacrolimus solutions used to treat each group of corneas are shown on the left. The first panel shows DAPI staining. Viable cells are stained green (second panel), while dead cells are stained red (third panel). The fourth panel shows a merged image of the images in the same row. Each image is a representative of three replicates (*n* = 3). All images were captured using ×10 magnification using a laser scanning confocal microscope.

Using the triple stain technique to assess corneal cell viability revealed that the viability of the used corneas was high, as evidenced by the intense green fluorescence in all corneas (panel 2, Figure3). However, the folds of the epithelium always contained a population of dead cells (panel 3, Figure 3). It is to be noted that no significant changes in fluorescence intensity could be observed in corneas treated in with 0.005% (*p* = 0.61), 0.1% (*p* = 0.11), or 0.2% (*p* = 0.06) tacrolimus solution or those treated with vehicle only (PBS), showing minor dead cells on the epithelial folds. Treatment with 0.01% and 0.05% tacrolimus solution showed an increase in staining intensity on and around the folds compared to control-treated corneas. In corneas treated with 0.1%, rare scattered dead cells were observed and the folds on corneal surfaces were viable, unlike concentrations higher than 0.1% and lower than 0.05% (Figure 3). The observed results indicated no significant cell death in response to tacrolimus eye suspension treatment.

### DAPI Staining

**FIGURE 4:**
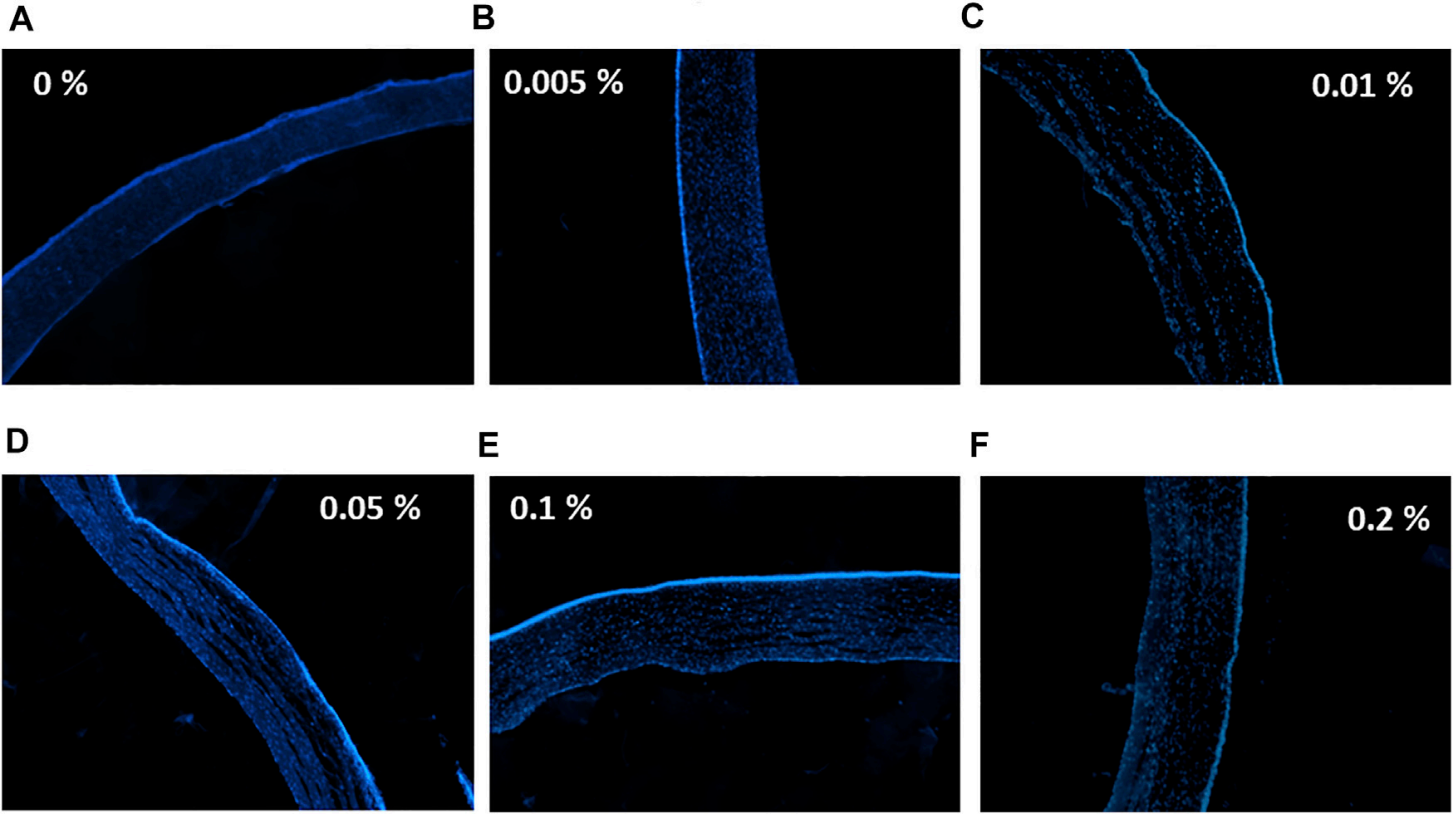
The effect of tacrolimus on stromal and epithelial cell densities. Concentrations of tacrolimus solutions used to treat each group of corneas are shown on the left. DAPI staining is shown in blue. **(A)** Control corneas treated with PBS, **(B)** corneas treated with 0.005% tacrolimus, **(C)** corneas treated with 0.01% tacrolimus, **(D)** corneas treated with 0.05% tacrolimus, **(E)** corneas treated with 0.1% tacrolimus, **(F)** corneas treated with 0.2% tacrolimus. Each image is a representative of three replicates (*n* = 3). All images were captured using ×10 magnification using a laser scanning confocal microscope.

Nuclei of epithelial and stromal cells were labeled with DAPI staining. Corneas treated with 0% (PBS only) and 0.005% tacrolimus solution showed intense staining of the epithelium and the highest population of viable stromal cells. However, treatment with 0.01%, 0.05%, 0.1%, and 0.2% concentrations showed a normal population of the stroma with keratocytes and good intensity and distribution of epithelial layers, as shown in Figure 4. Comparing corneas treated with tacrolimus solution, 0.005%, 0.01%, 0.05%, 0.1%, and 0.2% concentrations showed no significant changes in fluorescence intensity compared to control PBS treated corneas (*p* = 0.89, 0.13, 0.15, 0.31, and 0.18, resp.). The epithelial staining in corneas treated with tacrolimus *ex vivo* was comparable to negative controls (vehicle: PBS). The epithelium showed a regular distribution of cells and a normal number of layers (3–6 layers). Stromal cells in tacrolimus-treated human corneas were concentrated away from the epithelium and segregated near the endothelial layer. The density of stained cells in the epithelium was unchanged compared to PBS treated corneas (Figure 4).

## Tacrolimus Clinical Use for Severe Vernal Keratoconjunctivitis

### Case 1

**FIGURE 5:**
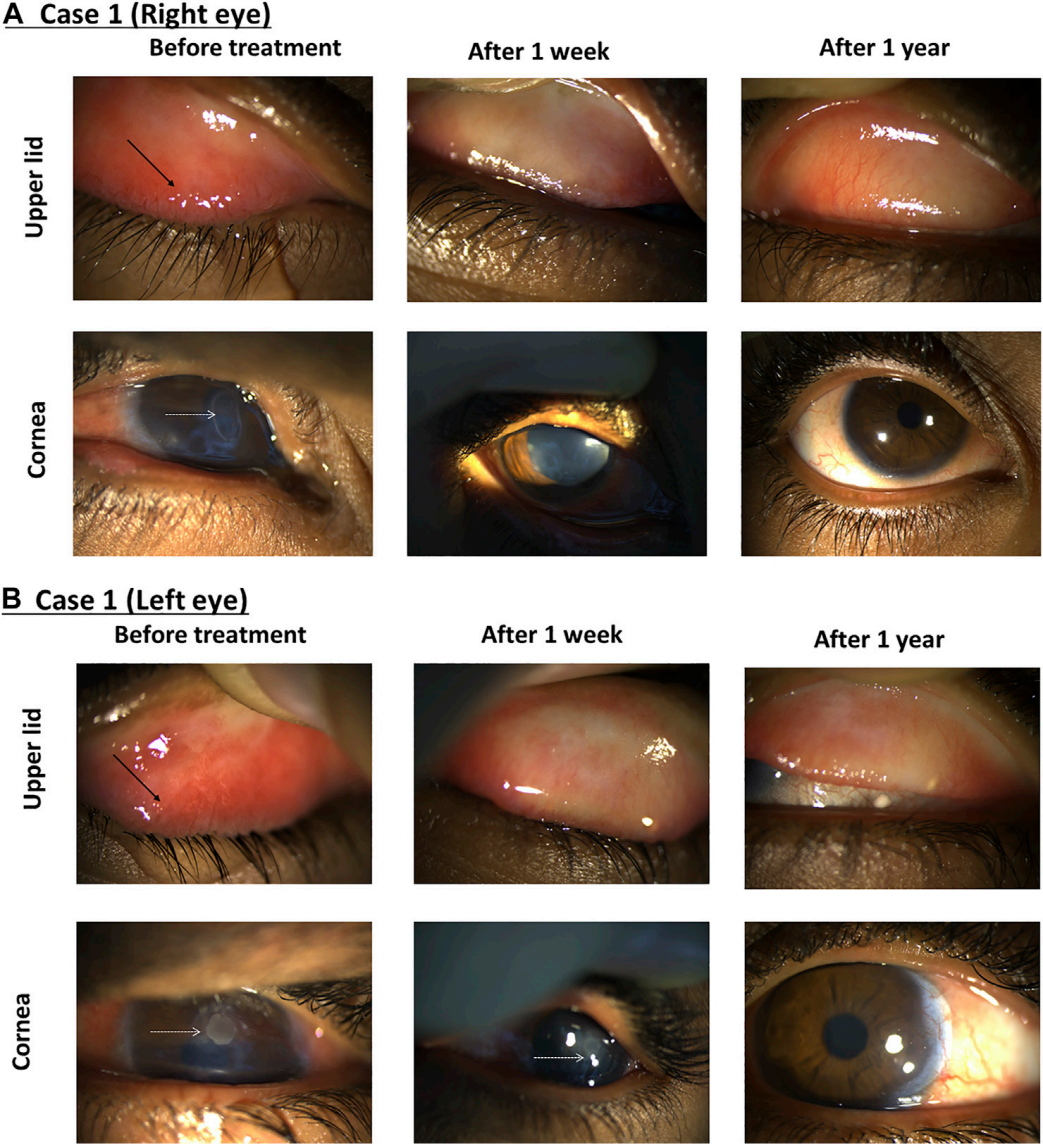
Using tacrolimus for a case of severe VKC. The right eye **(A)** and left eye **(B)** show the upper lid (top row) and the cornea (bottom row). Images taken from the first visit (left column), after 1 week (middle column) and 1 year (right column). Black arrows indicate conjunctival papillae, and white dotted arrows show shield ulcers.

A 13-year-old male patient presented with redness and pain in both eyes. Initial findings included bilateral large tarsal conjunctival papillae, bilateral peri-limbal injection, limbal conjunctival hypertrophic, and bilateral shield ulcers (Figure 5). The diagnosis was severe VKC of the mixed sub-type associated with bilateral shield ulcers, which were larger and worse on the left eye, associated with corneal neovascularization in the left eye and bilateral keratoconus. The patient was photophobic and experienced difficulties in opening his eyes. After obtaining informed consent from the patient’s family, bilateral corneal scraping was performed using an aseptic technique to remove the mucus plaque and was prescribed 0.03% tacrolimus and gatifloxacin eye drops four times daily. Two weeks later, the VKC was semi-active in both eyes. The shield ulcer totally resolved in the right eye, and there was significant improvement of the subepithelial stromal haze and total corneal epithelial healing in the left eye. The tacrolimus dose concentration was increased to 0.05% four times daily for both eyes in addition to tacrolimus ointment 0.1% at bedtime for both eyes. A month later, his VKC was inactive. Accordingly, the patient was admitted to the hospital for corneal collagen cross-linking in both eyes under general anesthesia to halt the progression of keratoconus. Additionally, 0.01 cc of subconjunctival triamcinolone acetonide was applied at the area of corneal neovascularization. The outcome was initially favorable. However, 6 months later, persistent but fine corneal vascularization was still noted in the left eye with progressive keratoconus in both eyes evident by continued steepening of the cornea by > 1 diopter in both eyes and a further drop of best-corrected visual acuities in both eyes. Consequently, corneal collagen cross-linking was repeated. Seven months postoperatively, there were no signs of progression of keratoconus, and the VKC remained inactive with tacrolimus 0.05% eye drops three times daily and tacrolimus ointment 0.1% Qhs. A year later, both eyes were healthy, with slight signs of fibrosis in the left upper tarsal conjunctiva in addition to a persistent very faint corneal haze and small-caliber corneal neovascularization in the left eye as a sequel of the initial large shield ulcer on presentation (Figure 5).

### Case 2

**FIGURE 6:**
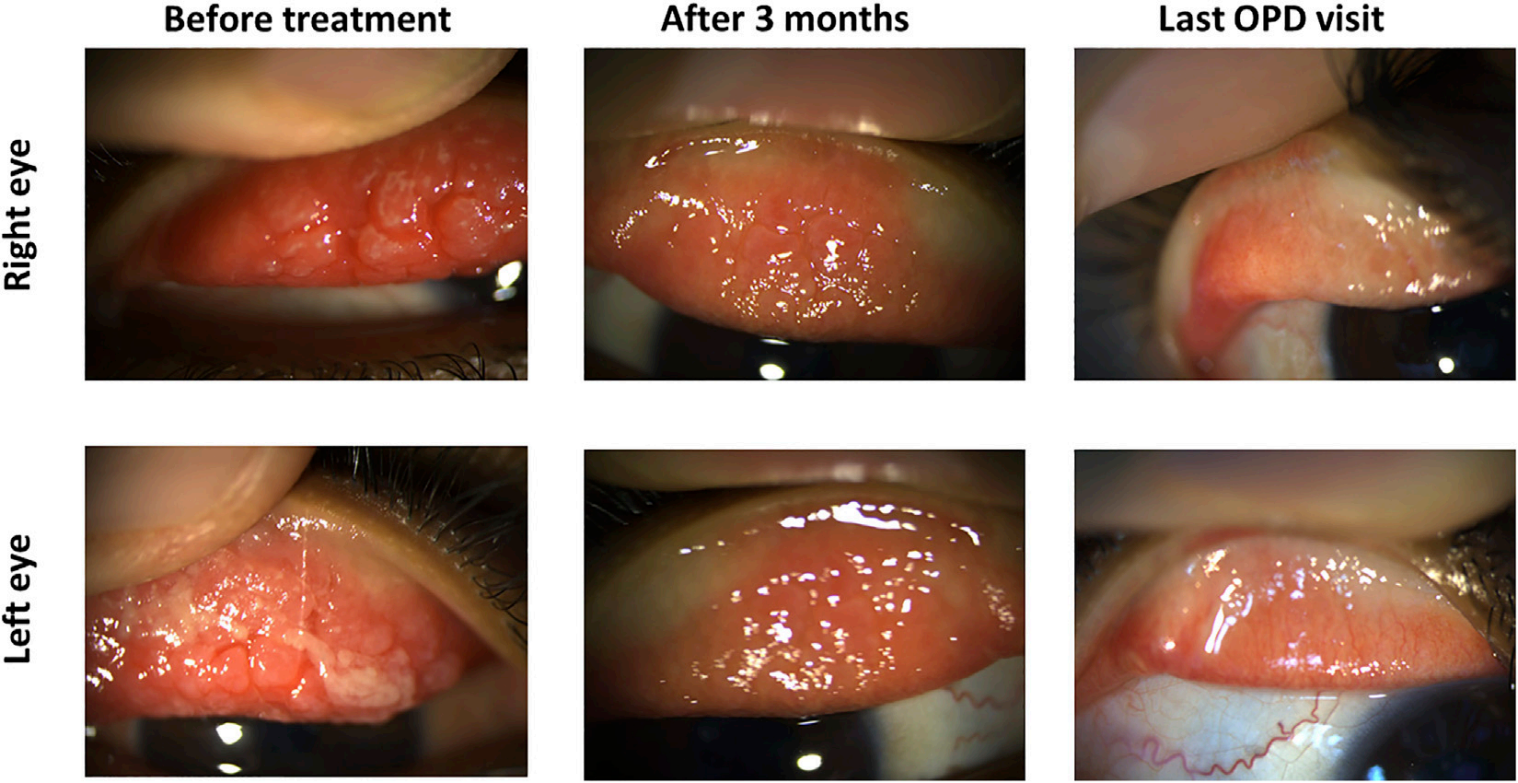
Tacrolimus eye suspension for Case 2 of VKC. The left panel shows the upper eyelid of the right and left eyes in the first visit before treatment with tacrolimus. Pictures from the next visit after 3 months are shown in the middle panel. The final outpatient department visit (OPD) after 6 months is shown in the left panel.

An 8-year-old male had a severe VKC tarsal type in both eyes. Bilateral cobble stone giant papillae with thick ropy mucus discharge were noted. He was given 0.03% tacrolimus eye drops four times daily. After 3 months, the inflammation was significantly reduced in both eyes, so tacrolimus ointment 0.1% was added before bedtime. Hence, the size of the papillae was further reduced within a month, and the patient was maintained on tacrolimus 0.03% eye drops three times daily and tacrolimus 0.1% ointments at bedtime (Figure 6).

### Case 3

**FIGURE 7:**
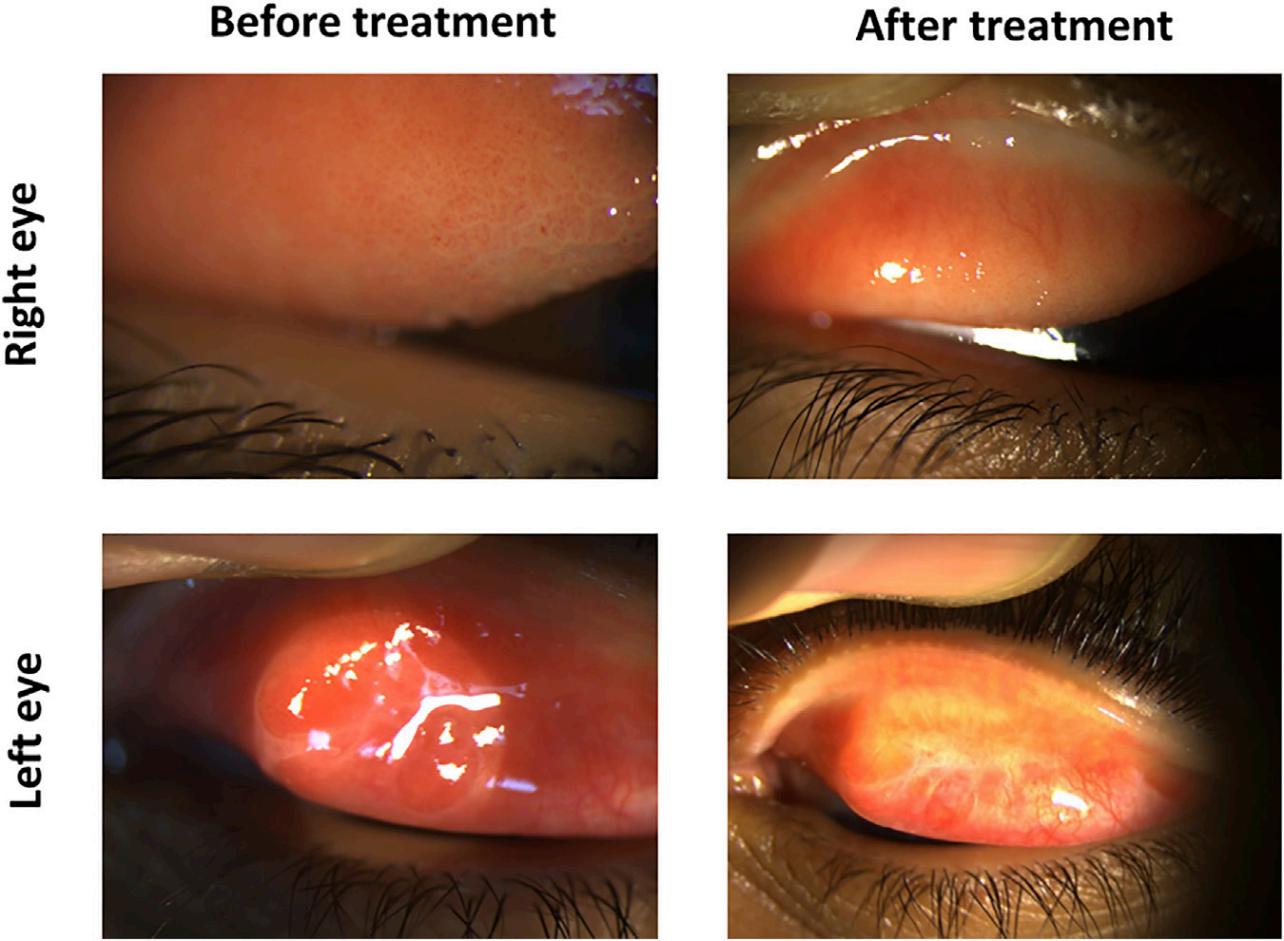
Tacrolimus eye suspension for Case 3 of VKC. The left panel shows the upper eyelid of the right and left eyes in the first visit before treatment with tacrolimus. Pictures from the final OPD visit after 5 months are shown in the left panel.

A 15-year-old male presented with severe itching. The patient history showed previous corneal collagen cross-linking in his right eye and cornea transplantation in this left eye for keratoconus. Ocular examination revealed moderate-sized tarsal conjunctival papillae in the right eye and large-sized tarsal conjunctival papillae associated with subconjunctival scarring in the left eye. The patient was given 0.03% tacrolimus eye suspension four times daily (QID) for both eyes. Three months later, the inflammation was subsided in the right eye and reduced in the left eye. Hence, the tacrolimus concentration was increased for the left eye to 0.05% QID and maintained at 0.03% QID to the right eye. Two months later, the inflammation equally subsided in both eyes, leaving some fibrotic changes in the left tarsal conjunctiva, a consequence of the previously chronic inflammation (Figure 7).

## Discussion

In this work, tacrolimus was investigated for its potential effect and the optimal concentration for the treatment of VKC. Topical administration of tacrolimus eye drops did not have a significant effect on human corneal thickness, with the concentration of 0.1% showing the minimal changes in this context. Tacrolimus did not result in swelling of the cornea at any of the tested concentrations, and the viability of the epithelium was good at all tested concentrations, with a few dead cells on the outermost layer of the epithelium. The viability of corneas at all tested concentrations was comparable to negative controls. However, it is worth noting that corneas treated with 0.1% or 0.05% concentrations showed almost no dead cells, indicating that corneal viability was best at these two concentrations. 0.1% tacrolimus-treated corneas showed the highest stromal cell density, indicating viable and healthy stroma. At all the tested concentrations, tacrolimus did not show signs of toxicity.

VKC is an atopic, serious, uncommon juvenile ophthalmological condition that affects the ocular surface and subsides by puberty. However, if it was improperly treated, it could result in corneal scars and vision loss over time. For several ocular allergic and inflammatory diseases, including VKC, the first-line therapy is usually antihistamines, mast stabilizing agents, or a combination of both. In contrast, corticosteroids are used as a last choice. This can be explained by the major role of inflammation that contributes to the symptoms of VKC, affecting their severity and persistence. However, it is to be mentioned that other factors may also play a role in VKC pathogenesis and other ophthalmic disorders as well such as neuronal factors, endocrine factors, and the involvement of specific immune cells (Sacchetti et al., 2021). Tacrolimus is one of the potent immunosuppressant drugs recently tested as therapeutic agents in several clinical conditions and showed promising, positive therapeutic outcomes. The safety of tacrolimus was evaluated based on the observed clinical signs and symptoms after administration, but the underlying cellular and molecular changes were not illustrated in the human cornea. The utilization of eye bank-donated human corneas in an *ex vivo* model provides a reliable tool for tracking the cellular and morphological changes taking place in the cornea upon topical administration of the drug, providing data superior to the data that would be obtained using *in vitro* models. The use of the *ex vivo* model in the current work also enables a more concise investigation of acute toxicity signs of topically applied tacrolimus, which serves as an indicator of its chronic toxicity effects. However, a major disadvantage of the *ex vivo* model is that isolated human corneas are deprived of an immune response, which renders *in vivo* testing inevitable for investigating the inflammatory process in VKC as reported before. As a common practice, dose frequencies in this research were increased over a short period of time and the signs of vitality were followed up on the short term. In the literature, a range of concentrations of tacrolimus ointments and eye drops was reported. A concretion of 0.03% tacrolimus eye drops in olive oil was used to treat Sjögren’s syndrome dry eye twice daily for 3 months, where tear film stability showed an improvement in diagnostic signs after 90 days (Moscovici et al., 2012). In another report, a significant improvement in the signs and symptoms of refractory VKC was observed after application of topical 0.005% tacrolimus eye drops on 10 patients (Kheirkhah et al., 2011). Another research group used a concentration of 0.1% to treat VKC and atopic keratoconjunctivitis successfully (Vichyanond et al., 2004; Ohashi et al., 2010). A preparation of 0.3% tacrolimus concentration was also used to successfully treat giant papillary conjunctivitis (Kymionis et al., 2008). Despite the reported positive effects of topical tacrolimus treatment, it is shown to be less effective than systemic treatment (Iwamoto et al., 1999). In an experimental rat model, oral doses were reported to range from 0.1 and to 10 mg/kg of body weight (Kawashima et al., 1988).

Investigating the safety of 0.1% suspension eye drops in a rabbit model of corneal transplantation was one of the few reports to address histological changes in tacrolimus-treated corneas, where the infiltration of inflammatory cells, neovascularization, and stromal thickening were used as parameters to assess the drug safety (Yuan et al., 2012). A concentration of 0.3% eye ointment twice daily for up to 14 months was shown effective (Rikkers et al., 2003).

In the current study, a range of concentrations was assigned to cover the previously tested concentrations and allocate the best concentration. The dosing frequency was increased to five times within 8 h to study the viability of human corneas under excessive tacrolimus treatment.

In the previously mentioned research reports, the drug improved clinical symptoms, but cytotoxicity data, molecular events, and long-term safety data for topical treatment were insufficient. Ocular irritation and inflammatory signs were the main parameters investigated in the literature regarding tacrolimus efficiency. Previous clinical studies employed Schirmer I test, break-up time (BUT), corneal fluorescein, and rose bengal staining to evaluate the efficiency of tacrolimus in the dry eye syndrome (Moscovici et al., 2012; Moscovici et al., 2015). In Case 2 mentioned in the current work, the treatment of a severe case of VKC was attempted starting with a concentration of 0.03% after ensuring that tacrolimus eye drops did not cause significant cell damage to the cornea. The concentration was later increased to 0.05% with the addition of tacrolimus ointment 0.1% at bedtime according to the clinical response of the disease to further enhance the recovery of the eye and resolution of the inflammation. Despite the observed positive results, it would also be interesting to test tacrolimus in a different dosage form, such as lipid nanoparticles, which can improve absorption and drug delivery (Puglia et al., 2021). It is noteworthy that many drug candidates intended for ophthalmological use were not tested on human corneas prior to clinical trials (Fogagnolo et al., 2020; Toro et al., 2021). Therefore, the currently utilized protocol in this work, where the *ex vivo* results were combined with the clinical testing of tacrolimus for severe VKC, is proposed as a safe and effective substitute for the use of corticosteroids. However, further investigations are required, including a more in-depth investigation of the molecular effect of tacrolimus on the eye. Additionally, the clinical testing part of the study should be performed on a larger number of patients to ensure the efficacy and safety of the treatment.

In order to assess the ocular irritation symptoms of dry eye disease, some reports used questionnaires like the Ocular Surface Disease Index (OSDI). Few research groups utilized molecular-based techniques such as flow cytometry to detect CD4 positive T cells in rat corneas to address the safety of tacrolimus at the molecular level (Iwamoto et al., 1999). However, in these reports, the inflammatory status was the only objective.

Regarding viability, in the current work, corneas looked healthier after 0.1% tacrolimus treatment as dead cells were almost undetectable in these corneas compared to those under other tested concentrations. The treatment with this concentration showed the least change in corneal thickness. In addition, at 0.1% tacrolimus, the stroma showed a high cellular population, and the epithelial layers looked healthy and comparable to normal. It is therefore recommended to use this concentration for ocular use of tacrolimus. A Draize test is required as a first step to obtain an approval for the use of tacrolimus for this purpose.

In conclusion, topical administration of tacrolimus was not toxic at all tested concentrations, and the 0.1% concentration has shown the best corneal viability. A concentration range of tacrolimus was shown to be safe and effective for use in severe cases of VKC and is proposed as a topical ocular immunosuppressant drug enabling clinicians to incrementally increase the drug concentration in accordance with the clinical severity of the disease to achieve the optimal therapeutic response.

## Data Availability

The original contributions presented in the study are included in the article/Supplementary Material. Further inquiries can be directed to the corresponding author.
